# Prospective Association between Smartphone Addiction and Perceived Stress and Moderation of Boredom during COVID-19 in China

**DOI:** 10.3390/ijerph192215355

**Published:** 2022-11-21

**Authors:** Jiankang He, Xue Yang, Mingxuan Du, Chengjia Zhao, Xin Wang, Guohua Zhang, Honglei Peng

**Affiliations:** 1 Department of Psychology, School of Mental Health, Wenzhou Medical University, Wenzhou 325035, China; 2 Center for Health Behaviours Research, JC School of Public Health and Primary Care, Faculty of Medicine, The Chinese University of Hong Kong, Hong Kong 999077, China; 3 School of Education, Renmin University of China, Beijing 100872, China; 4 The Affiliated Kangning Hospital, Wenzhou Medical University, Wenzhou 325035, China; 5 The Audit Office, Wenzhou Medical University, Wenzhou 325035, China; 6 School of Public Health and Management, Wenzhou Medical University, Wenzhou 325035, China

**Keywords:** college students, smartphone addiction, perceived stress, boredom, COVID-19 pandemic

## Abstract

Smartphone addiction (SPA) is prevalent in college students and harms their healthy development, and perceived stress (PS) has been a well-documented risk factor of SPA. People often experienced boredom during COVID-19; however, its effect on behavioral/mental health during the pandemic has been rarely tested. We investigated the prospective association between SPA and PS before and during COVID-19, as well as the moderation of boredom. A total of 197 college students participated in four-wave surveys from December 2018 to June 2020 in China. The cross-lagged model was developed to investigate the prospective association between SPA and PS from T1 to T4. Boredom was added to the model at T4 as a moderator to explore the moderating role of boredom during COVID-19. The results showed that the pandemic changed PS’s prediction on SPA. During COVID-19, boredom significantly affected SPA and PS and moderated the link from PS at T3 to PS at T4. The results suggest that the prospective associations between SPA and PS varied before and during COVID-19. Prevention of SPA should be conducted for new students and should be used to enhance their stress coping capacity. Intervention programs for eliminating boredom may be effective for reducing stress and SPA during COVID-19.

## 1. Introduction

Smartphone use is popular among college students and smartphone addiction (SPA) is prevalent in this age group [1,2]. In China, SPA is generally recognized as excessive use of smartphones and/or out of control smartphone use, which leads to impaired psychological and behavioral function [3]. Although SPA can be characterized by withdrawal symptoms, tolerance, dependence, and social problems as a consequence of overusing smartphones [4], due to a lack of pathological basis, it is not yet seen as a behavioral addiction and there is no universally used term [5]; it can also be known as mobile phone dependence and problematic smartphone use [5,6]. A high prevalence of SPA has been reported among students, ranging from 40.6% to 67.0% [7,8,9,10]. The negative consequences of SPA on students include, but are not limited to, increased loneliness and physical injuries, as well as decreased relationship quality, sleep quality, and GPA [9,11], and even cognitive failure [12].

Individuals will perceive stress when they lack sufficient resources to address situations that are perceived as demanding or threatening [13]. College students have to face many adaptation issues and challenges, such as academic completion, interpersonal communication, and career planning and employment, which may result in college students feeling great pressure [6]. Based on the General Strain Theory, individuals use a variety of coping strategies to relieve tension and eliminate its negative consequences [14]. The smartphone is an important and handy means for college students to cope with stress, given its access to films, short videos, gaming, and social networking [15,16,17]. Meanwhile, previous studies have extended the application of this theory to longitudinal causality. For example, based on the General Strain Theory and the longitudinal mediation model, Hautala and Sittner [18] found that perceived racial discrimination predicts delinquency; Cho and Galehan [19] confirmed the longitudinal effect of stress events (i.e., bullying victimization, child abuse, and peer conflict) on delinquency. Furthermore, Yang et al. [20] confirmed that this theory can be applied to stress and internet use, and stress is an important antecedent of internet addiction [14]. Thus, perceived stress (PS) may lead to excessive use of a smartphone and ultimately cause SPA. Cross-sectional studies have shown that SPA was significantly associated with PS in college students [7,21], but few longitudinal studies have explored the causality [22]. This longitudinal study aims to inquire about the development trends and interrelationship of college students’ SPA and PS.

At the end of 2019, a pandemic emerged and SARS-CoV-2 spread rapidly around the world. This epidemic then became a public health emergency of global concern in 2020 [23]. As a means of disease control, China’s Ministry of Education postponed the start of the spring semester in 2020. Prolonged stay-at-home isolation, reduced outdoor activity, and altered daily routines became new sources of stress for students [24]. Numerous studies found that emotional problems, such as depression and anxiety, increased significantly among college students during COVID-19 [24,25]. Boredom became a common emotion perceived by students during the epidemic [26], which refers to the aversive experience of being unable to engage in satisfying activities, including being unable to focus on internal and external information or activities [27].

The Compensatory Internet Use Theory points out that when individuals lack social stimuli and outdoor activities, they will go online to seek compensation or sensation [28,29]. As a result, isolation during the pandemic may have enhanced the motivation to use smartphones; however, over-reliance on electronic contact to kill time, escape real-life problems, or mitigate negative emotions may lead to SPA [16]. A meta-analysis showed the prevalence of SPA during the pandemic was around 30.7% [30], and a study found that in China, 46.8% of participants reported an increased dependence and time spent on the internet during COVID-19 [31]. These findings were related to age, motives, illness fears, anxiety, exhaustion, academic procrastination, quality of life, and so on [32,33,34]. Based on this, we thought that the pandemic may change the development trends and interrelationship of SPA and PS. Meanwhile, some studies have shown that boredom was associated with SPA and PS before and during the COVID-19 pandemic [35,36,37]. Participants reported that boredom was a stressor during COVID-19 [38,39] and boredom may play a role in the above relationships. Furthermore, a study found that boredom was a moderator between students’ interactions, including student–student interactions, student–instructor interactions, and student–content interactions, as well as online learning persistence [40]; moreover, Yang et al. [41] reported that boredom moderated the relationship between attention control and SPA. Thus, boredom can be seen as a moderator; however, we found that there was no study on boredom’s moderating effects on the changes in SPA and PS and their associations over time.

So far, there has been no research on the development trends and interrelationship of SPA and PS. This study aimed to fill these research gaps by conducting a longitudinal study. Furthermore, after the outbreak of COVID-19, there were no studies about boredom and its role on SPA and PS based on a longitudinal study. Thus, we hypothesized that:

**Hypothesis 1** **(H1).** *College students’ SPA and PS would change to a certain extent before and during COVID-19;*

**Hypothesis 2** **(H2).** *PS would have a significant predictive effect on SPA over time;*

**Hypothesis 3** **(H3).** *Boredom during COVID-19 would moderate the changes in SPA and PS and their associations.*

## 2. Materials and Methods

### 2.1. Data Collection

The sample of this study was a convenience sample from classroom settings. The college students of Wenzhou Medical University in Zhejiang Province, China, participated in this study. By consulting these participants’ counselors, we chose the time periods when the participants had no scheduled classes or exams so that the questionnaires could be easily implemented. At baseline (December 2018; T1), freshmen were invited into this study and its subsequent surveys were carried out in June 2019 (T2), December 2019 (T3), and June 2020 (T4), respectively. Firstly, a research assistant introduced this study to the participants and provided the informed consent forms. Students’ student identity documents (ID) were recorded to match the longitudinal data. We did not record their names and researchers could not identify students’ names from their answers or student IDs. Therefore, this study was anonymous. Then, researchers promised the confidentiality of data and that only the researchers could access their data. Participants could exit the study and seek help from the researchers at any time. Finally, each participant received 20 Chinese Yuan (about 2.94 USD) at every turn. The Ethics Committee of the Wenzhou Medical University approved this study.

At T1, 212 college students finished all questionnaires, and 197 (92.92% of the 212) completed the subsequent surveys at T2, T3, and T4. The reasons for sample loss included quitting and changes in majors. We used the data from the college students who completed all four surveys for the analysis. Of the 197 participants, 81 (41.12%) were male; 103 (52.28%) came from an urban environment and 94 (47.72%) came from a rural environment; 95 (48.22%) came from a one-child family; and 28 (14.21%) majored in anesthesia, 29 (14.72%) majored in forensic medicine, 55 (27.92%) majored in stomatology, and 85 (43.15%) majored in Chinese Medicine.

### 2.2. Measures

#### 2.2.1. Smartphone Addiction

SPA was assessed by the Mobile Phone Addiction Index (MPAI) [42] and the Chinese version was developed by Liu and Wang [43]. The 17 items of MPAI were rated on Likert scales (from 1 = never to 5 = always). The sum of scores ranged from 17 to 85, and higher score indicated higher SPA tendencies. MPAI has good validity and reliability among college students in China [44]. In this study, Cronbach’s α for MPAI were 0.85, 0.88, 0.91, and 0.76 from T1 to T4, respectively.

#### 2.2.2. Perceived Stress

PS was evaluated by the Perceived Stress Scale (PSS) [13]. Yang and Huang developed its Chinese version [45]. The 14 items of the PSS were rated on Likert scales (from 1 = never to 5 = very often). The sum of scores ranged from 14 to 70. A higher total score represented a stronger intensity of PS. The PSS has good validity and reliability in the Chinese population [46]. In this sample, the Cronbach’s α for PSS were 0.80, 0.83, 0.86, and 0.69 from T1 to T4, respectively.

#### 2.2.3. Boredom

We used the Multidimensional State Boredom Scale (MSBS) [47] to assess boredom. Three items from the Chinese version of the MSBS [48] were adapted to assess boredom at T4 during the pandemic (i.e., “I feel bored during COVID-19”, “I am easily distracted during COVID-19”, and “Time is passing by slower than usual during COVID-19”). The items were rated on Likert scales (from 1 = strongly disagree to 5 = strongly agree). The sum of scores ranged from 3 to 15 and a higher score indicated a higher level of boredom. The items were already used in Chinese studies [26]. In this sample, the Cronbach’s α for MSBS was 0.67.

### 2.3. Statistical Methods

Descriptive analyses, significance tests of difference, and Pearson’s correlation analyses were executed by IBM SPSS statistics 26.0 (IBM Corporation, Armonk, NY, USA). Furthermore, Amos (Amos 24.0) (IBM Corporation, Armonk, NY, USA) was used to test the cross-lagged model and moderation effect. The package *semPower* 1.2.0 in R 4.2.2 (R Core Team, Vienna, Austria) was used to do the a priori test [49]. Firstly, descriptive analyses and a one-way analysis of variance (ANOVA) were performed to analyze the characteristics of the participants. Then, repeated measurement ANOVA was used to compare the levels of SPA and PS from T1 to T4. Secondly, the Pearson correlation coefficient was used to inspect the correlations between SPA, PS, and boredom over time. Thirdly, based upon structural equation modeling, cross-lagged modeling analysis was conducted to examine the relationships among SPA and PS at the four time points. Then, the boredom variable was added on the basis of the cross-lagging model to evaluate its moderating effects between SPA and PS at T3 and T4. All models used the maximum likelihood estimation, and model fits were evaluated by the following indices: the Chi-square (χ^2^) goodness of fit statistic, degree of freedom (*df*), the root mean square error of approximation (RMSEA), the Tucker–Lewis index (TLI), and the Comparative Fit Index (CFI). It was considered acceptable when RMSEA ≤ 0.08, TLI ≥ 0.90, and CFI ≥ 0.90 [50]. Thus, this suggested that our study needed at least 294 samples per time for the cross-lagged model (RMSEA = 0.08, alpha error = 0.05, power = 0.8, *df* = 8), and needed 165 samples for the moderation model (RMSEA = 0.08, alpha error = 0.05, power = 0.8, *df* = 20). In this study, *p* < 0.05 was considered statistically significant.

## 3. Results

### 3.1. Preliminary Analyses

The results of Harman’s single factor test showed that without rotation, 34 principal components were extracted. and the first variance contribution rate was 18.95%; this was lower than 40.0%, which was considered the critical value [51]. Therefore, this study had no serious common method bias. We compared those who finished all four surveys (N = 197) versus those who missed at T2 and T3 (N = 15). There was no significant difference in sociodemographic characteristics or the levels of the measured variables in the two groups (*p* > 0.05).

The time effects were significant for both SPA and PS [*F*_(2.90,567.98)_ = 4.79, *p* = 0.003, η^2^*_p_* = 0.02; *F*_(3,588)_ = 12.97, *p* < 0.001, η^2^*_p_* = 0.06]. The results of the post-hoc tests showed that the levels of SPA at T2 (95% CI: 0.20–3.08, *p* = 0.026), T3 (95% CI: 0.67–3.76, *p* = 0.005), and T4 (95% CI: 1.06–4.04, *p* = 0.001) were significantly lower than T1, and there was no notable difference between T2, T3, and T4. Meanwhile, the levels of PS at T2 (95% CI: 1.43–3.12, *p* < 0.001), T3 (95% CI: 1.27–3.08, *p* < 0.001), and T4 (95% CI: 1.70–3.47, *p* < 0.001) were significantly lower than T1, and there was no notable difference between T2, T3, and T4. Thus, H1 was supported.

As shown in Table 1, SPA and PS at the four time points and boredom at T4 were significantly and positively associated with each other.

### 3.2. Cross-Lagged Modeling

**Figure 1:**
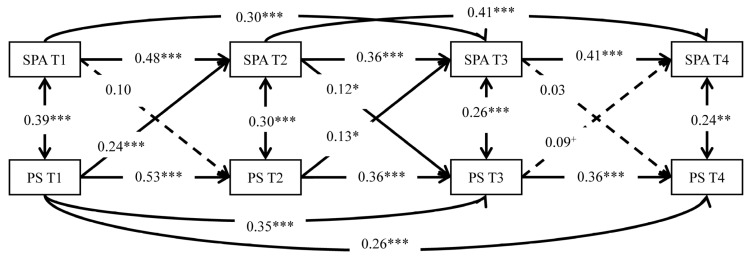
The cross-lagged model among smartphone addiction (SPA) and perceived stress (PS) with standardized path coefficients (β). Note: Dashed lines indicate non-significant paths. N = 197. ^+^ *p* < 0.10, * *p* < 0.05, ** *p* < 0.01, *** *p* < 0.001. The same below.

Although the sample size was limited, the model was acceptable (χ^2^ = 16.43, *df* = 8, *p* = 0.037, RMSEA = 0.07, TLI = 0.96, CFI = 0.99). Figure 1 shows that PS at T1 significantly and positively predicted SPA at T2 (β = 0.24, *p* < 0.001), PS at T2 significantly and positively predicted SPA at T3 (β = 0.13, *p* = 0.032), and SPA at T2 significantly and positively predicted PS at T3 (β = 0.12, *p* = 0.036). Thus, H2 was supported at T1–T3, but was not supported at T3–T4.

### 3.3. The Moderating Role of Boredom at T4

**Figure 2:**
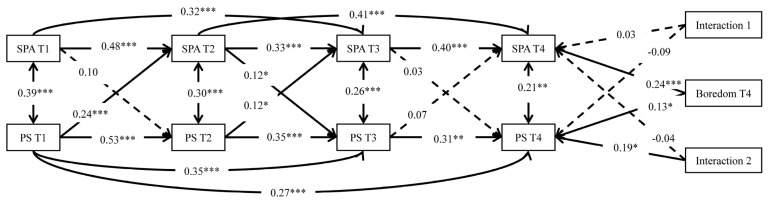
The moderated cross-lagged model of smartphone addiction (SPA), perceived stress (PS), and boredom (N = 197). Note: Interaction 1 = Boredom at T4 × SPA at T3; Interaction 2 = Boredom at T4 × PS at T3.

The model with moderating variables was also acceptable (χ^2^ = 38.21, *df* = 20, *p* = 0.008, RMSEA = 0.07, TLI = 0.94, CFI = 0.98). Figure 2 shows the main effects of boredom on SPA (β = 0.24, *p* < 0.001) and PS at T4 (β = 0.13, *p* = 0.036). Furthermore, the moderating effect of boredom on the association between PS at T3 and PS at T4 was notable (β = 0.19, *p* = 0.013). Therefore, H3 was partially supported.

**Figure 3:**
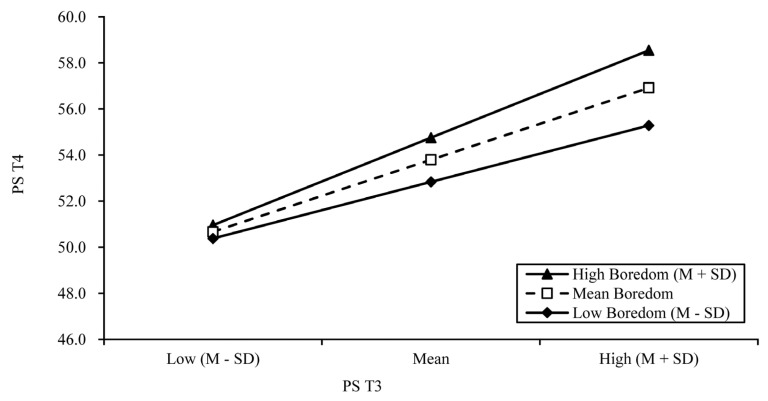
The moderating effect of boredom at T4 on the association between PS at T3 and PS at T4 (N = 197).

We conducted a simple slope test to assess the moderating role of boredom at T4 between PS at T3 and PS at T4 (Figure 3). The positive relationship between PS at T3 and PS at T4 was stronger when the level of boredom was higher (β = 0.58, b = 0.48, *p* < 0.001) than that when the level of boredom was low (β = 0.38, b = 0.31, *p* < 0.001).

## 4. Discussion

### 4.1. College Students’ SPA and PS before and during COVID-19

This study showed that college students had higher levels of SPA and PS at baseline than those at follow-up. Freshmen’s adaptability may be the key factor, which could affect the mental health and SPA of college students [52,53]; this may be because participants just started their college life and were adjusting to new challenges with limited coping resources when the baseline survey was conducted [6]. It is consistent with prior studies, which report that college students’ stress would decrease over time [54]; however, it is not consistent with the COVID-19 studies, which argue that SPA and PS increased during COVID-19 [55,56]. It is possible that this was because the pandemic was under control in China when the survey was conducted at T4 [57] and participants had adapted to the stress related to the COVID-19. Our findings highlight the importance of support programs for college students to facilitate their adaptation to college life. Furthermore, for college students, their connections with teachers, schoolmates, and the school overall are important for alleviating the risks of smartphone addiction [58]. School administrators should pay more attention to it. Furthermore, for medical students, being limited at home could affect their internships, academic activities, and so on, although we did not collect this information. A follow-up study focusing on this issue is needed in the future.

### 4.2. Prospective Relationship between SPA and PS

In this study, the results showed that SPA was notably and positively correlated with PS over time in college students. Furthermore, according to the cross-lagged analysis, we found that PS at T1 and T2 significantly and positively predicted SPA at T2 and T3, respectively. This is in agreement with the General Strain Theory’s extension of application (stress is the antecedent of internet addiction) [14] and the results of the cross-sectional study [21], which suggests that PS was a risk factor for triggering SPA before the COVID-19 outbreak. Given this, when college students perceived stress, they were more likely to release their stress via smartphones. Furthermore, in the absence of examination of other behaviors in this study, we have no way of knowing the propensity for coping styles. On the other hand, SPA at T2 significantly predicted PS at T3, indicating that SPA may in turn increase PS [59]. Based on the compensation theory, this may be because using smartphones could allow for an escape from negative emotions but could not result in a change in real life. It is interesting that we found different relationships between SPA and PS before and during COVID-19. Their non-significant relationship during COVID-19 suggests that the causes of SPA or PS have changed and may have become the pandemic itself, rather than as a result of changes due to the pandemic. For example, the major motives for people excessively using smartphones during COVID-19 was to maintain their basic daily needs (e.g., social connection, information seeking, online shopping, online study). Furthermore, there may be other links between PS and SPA, such as PS moderating the association between motivation and SPA [46]. This also implies that the relationship between PS and SPA is complex and may vary across contexts.

### 4.3. Effects of Boredom during COVID-19

In both correlation and model testing, boredom at T4 was correlated with PS and SPA significantly and positively, which is consistent with the studies before COVID-19 [41,60] and during the pandemic [61]. In addition, boredom at T4 exacerbated the perception of stress during COVID-19, making students with stress more likely to perceive stress. Therefore, boredom reduction programs that can attract attention [41], such as social activities [62] and exercise [63], may help to mitigate students’ perceived stress; however, the results were not in full compliance with the Compensatory Internet Use Theory: i.e., boredom did not promote the development of SPA. This is an interesting phenomenon that deserves further exploration. A possible reason for this was that during the epidemic, college students were more inclined to social interaction [62] and physical exercise [63] when they felt bored, and thus did not significantly increase the excessive use of smartphones. Based on this theory, future research should pay attention to life events, such as changing of exercise routines, internships, and other activities among medical students. Colleges and universities can provide education programs and campaigns for the promotion of adaptive entertainment activities and healthy digital-use lifestyles to help students adapt to the pandemic and accept it as the new “normal”.

### 4.4. Limitations

Firstly, the generalizability of the results may be limited due to the small sample size and convenience sampling. Secondly, the Cronbach’s alpha (0.67) of the MSBS at T4 was acceptable but relatively low. Since we only used three items and adapted them into the context of the COVID-19 pandemic, they should be further validated in future studies. Thirdly, this study was based on self-report measures that may have induced report bias. Future works should consider multiple participants (e.g., family and friends) and experimental measurements of stress and smartphone use to collect data. Last, the data on boredom was cross-sectional. It is necessary to conduct longitudinal studies to validate the role of boredom during COVID-19, especially in other countries where there were not such strict restrictions as in China.

## 5. Conclusions

The four-wave longitudinal study is conducive to a better understanding of the prospective association between SPA and PS and the moderating role of boredom among Chinese college students during the COVID-19 pandemic. There existed a reciprocal relationship between PS and SPA before COVID-19, while this relationship did not remain during COVID-19. The roles of boredom in SPA and PS during COVID-19 should be of concern. Precautions for SPA and stress coping training should be provided for freshmen students. Furthermore, intervention programs for eliminating boredom may be effective for decreasing stress and SPA during the COVID-19 pandemic.

## Figures and Tables

**Table 1:** Pearson correlation coefficients, mean (M), and standard deviation (SD) for the measured variables (N = 197).

Variables	1	2	3	4	5	6	7	8	9
1. SPA T1	1								
2. SPA T2	0.57 ***	1							
3. SPA T3	0.56 ***	0.59 ***	1						
4. SPA T4	0.54 ***	0.70 ***	0.70 ***	1					
5. PS T1	0.39 ***	0.42 ***	0.38 ***	0.40 ***	1				
6. PS T2	0.31 ***	0.47 ***	0.39 ***	0.49 ***	0.57 ***	1			
7. PS T3	0.37 ***	0.44 ***	0.50 ***	0.47 ***	0.61 ***	0.61 ***	1		
8. PS T4	0.23 **	0.32 ***	0.31 ***	0.43 ***	0.50 ***	0.50 ***	0.54 ***	1	
9. Boredom T4	0.22 **	0.17 *	0.16 *	0.37 ***	0.17 *	0.20 **	0.19 **	0.25 ***	1
M	48.27	46.63	46.06	45.72	39.00	36.73	36.82	36.42	9.16
SD	10.57	11.58	12.66	11.57	6.00	6.84	8.00	6.52	2.43

* *p* < 0.05, ** *p* < 0.01, *** *p* < 0.001.

## Data Availability

The data presented in this study are available on request from the corresponding author. The data are not publicly available due to the privacy of participants.

## References

[1] Liu H., Yao D. (2016). The Relationship between Mobile Phone Addiction and Loneliness, Self-harmony of University Students. Stud. Psychol. Behav..

[2] Long J., Liu T.-Q., Liao Y.-H., Qi C., He H.-Y., Chen S.-B., Billieux J. (2016). Prevalence and correlates of problematic smartphone use in a large random sample of Chinese undergraduates. BMC Psychiatry.

[3] Liu Q., Yang Y., Lin Y., Yu S., Zhou Z. (2017). Smartphone Addiction: Concepts, Measurements, and Factors. Chin. J. Clin. Psychol..

[4] Choi S.-W., Kim D.-J., Choi J.-S., Ahn H., Choi E.-J., Song W.-Y., Kim S., Youn H. (2015). Comparison of risk and protective factors associated with smartphone addiction and Internet addiction. J. Behav. Addict..

[5] Panova T., Carbonell X. (2018). Is smartphone addiction really an addiction?. J. Behav. Addict..

[6] Zhang G., Yang X., Tu X., Ding N., Lau J.T.F. (2020). Prospective relationships between mobile phone dependence and mental health status among Chinese undergraduate students with college adjustment as a mediator. J. Affect. Disord..

[7] Lei L.Y.-C., Ismail M.A.-A., Mohammad J.A.-M., Yusoff M.S.B. (2020). The relationship of smartphone addiction with psychological distress and neuroticism among university medical students. BMC Psychol..

[8] Alageel A.A., Alyahya R.A., Bahatheq Y.A., Alzunaydi N.A., Alghamdi R.A., Alrahili N.M., McIntyre R.S., Iacobucci M. (2021). Smartphone addiction and associated factors among postgraduate students in an Arabic sample: A cross-sectional study. BMC Psychiatry.

[9] Alotaibi M.S., Fox M., Coman R., Ratan Z.A., Hosseinzadeh H. (2022). Smartphone Addiction Prevalence and Its Association on Academic Performance, Physical Health, and Mental Well-Being among University Students in Umm Al-Qura University (UQU), Saudi Arabia. Int. J. Environ. Res. Public Health.

[10] de Freitas B.H.B.M., Gaíva M.A.M., Diogo P.M.J., Bortolini J. (2022). Relationship between Lifestyle and Self-Reported Smartphone Addiction in adolescents in the COVID-19 pandemic: A mixed-methods study. J. Pediatr. Nurs..

[11] Li J., Lepp A., Barkley J.E. (2015). Locus of control and cell phone use: Implications for sleep quality, academic performance, and subjective well-being. Comput. Hum. Behav..

[12] He A., Xia Y. (2019). The Influence of Mobile Phone Addiction on Cognitive Failure in Undergraduates: A Moderated Mediation Model. Psychol. Dev. Educ..

[13] Cohen S., Kamarck T., Mermelstein R. (1983). A global measure of perceived stress. J. Health Soc. Behav..

[14] Jun S., Choi E. (2015). Academic stress and Internet addiction from general strain theory framework. Comput. Hum. Behav..

[15] Sohn S., Rees P., Wildridge B., Kalk N., Carter B. (2019). Prevalence of problematic smartphone usage and associated mental health outcomes amongst children and young people: A systematic review, meta-analysis and GRADE of the evidence. BMC Psychiatry.

[16] Huang Q., Chen X., Huang S., Shao T., Liao Z., Lin S., Li Y., Qi J., Cai Y., Shen H. (2021). Substance and Internet use during the COVID-19 pandemic in China. Transl. Psychiatry.

[17] Wu Y.-L., Lin S.-H., Lin Y.-H. (2021). Two-dimensional taxonomy of internet addiction and assessment of smartphone addiction with diagnostic criteria and mobile apps. J. Behav. Addict..

[18] Hautala D., Sittner K. (2019). Longitudinal Mechanisms Linking Perceived Racial Discrimination to Aggressive Delinquency among North American Indigenous Youth. J. Res. Crime Delinq..

[19] Cho S., Galehan J. (2019). Stressful Life Events and Negative Emotions on Delinquency Among Korean Youth: An Empirical Test of General Strain Theory Assessing Longitudinal Mediation Analysis. Int. J. Offender Ther. Comp. Criminol..

[20] Yang X., Lau J.T.F., Wang Z., Lau M.C.M. (2018). Potential roles of masculine role discrepancy, discrepancy stress, and self-esteem in affecting addictive use of social networking sites among Chinese men: A random population-based study. J. Behav. Addict..

[21] Kuang-Tsan C., Fu-Yuan H. (2017). Study on Relationship Among University Students’ Life Stress, Smart Mobile Phone Addiction, and Life Satisfaction. J. Adult Dev..

[22] Thomée S. (2018). Mobile Phone Use and Mental Health. A Review of the Research That Takes a Psychological Perspective on Exposure. Int. J. Environ. Res. Public Health.

[23] Timeline: World Health Organization’s COVID-19 Response. https://www.who.int/emergencies/diseases/novel-coronavirus-2019/interactive-timeline.

[24] Tull M.T., Edmonds K.A., Scamaldo K.M., Richmond J.R., Rose J.P., Gratz K.L. (2020). Psychological Outcomes Associated with Stay-at-Home Orders and the Perceived Impact of COVID-19 on Daily Life. Psychiatry Res..

[25] Ahmed M.Z., Ahmed O., Aibao Z., Hanbin S., Siyu L., Ahmad A. (2020). Epidemic of COVID-19 in China and associated Psychological Problems. Asian J. Psychiatry.

[26] Yang X., Hu H., Zhao C., Xu H., Tu X., Zhang G. (2021). A longitudinal study of changes in smart phone addiction and depressive symptoms and potential risk factors among Chinese college students. BMC Psychiatry.

[27] Eastwood J.D., Frischen A., Fenske M.J., Smilek D. (2012). The unengaged mind: Defining boredom in terms of attention. Perspect. Psychol. Sci..

[28] Kardefelt-Winther D. (2014). A conceptual and methodological critique of internet addiction research: Towards a model of compensatory internet use. Comput. Hum. Behav..

[29] Garrote G.P.D.A., Rubio L., Gómez B.M., Buedo-Guirado C. (2021). Smartphone Abuse Amongst Adolescents: The Role of Impulsivity and Sensation Seeking. Front. Psychol..

[30] Alimoradi Z., Lotfi A., Lin C.-Y., Griffiths M.D., Pakpour A.H. (2022). Estimation of Behavioral Addiction Prevalence During COVID-19 Pandemic: A Systematic Review and Meta-analysis. Curr. Addict. Rep..

[31] Sun Y., Li Y., Bao Y., Meng S., Sun Y., Schumann G., Kosten T., Strang J., Lu L., Shi J. (2020). Brief Report: Increased Addictive Internet and Substance Use Behavior During the COVID-19 Pandemic in China. Am. J. Addict..

[32] Albursan I.S., Qudah M.F.A., Al-Barashdi H.S., Bakhiet S.F., Darandari E., Al-Asqah S.S., Hammad H.I., Al-Khadher M.M., Qara S., Al-Mutairy S.H. (2022). Smartphone Addiction among University Students in Light of the COVID-19 Pandemic: Prevalence, Relationship to Academic Procrastination, Quality of Life, Gender and Educational Stage. Int. J. Environ. Res. Public Health.

[33] Popescu A.-M., Balica R., Lazăr E., Bușu V.O., Vașcu J.-E. (2022). Smartphone addiction risk, technology-related behaviors and attitudes, and psychological well-being during the COVID-19 pandemic. Front. Psychol..

[34] Wen F., Ding Y., Yang C., Ma S., Zhu J., Xiao H., Zuo B. (2022). Influence of smartphone use motives on smartphone addiction during the COVID-19 epidemic in China: The moderating effect of age. Curr. Psychol..

[35] Yan L., Gan Y., Ding X., Wu J., Duan H. (2021). The relationship between perceived stress and emotional distress during the COVID-19 outbreak: Effects of boredom proneness and coping style. J. Anxiety Disord..

[36] Yue H., Zhang X., Sun J., Liu M., Li C., Bao H. (2021). The relationships between negative emotions and latent classes of smartphone addiction. PLoS ONE.

[37] Zhao J., Ye B., Yu L. (2021). Peer Phubbing and Chinese College Students’ Smartphone Addiction During COVID-19 Pandemic: The Mediating Role of Boredom Proneness and the Moderating Role of Refusal Self-Efficacy. Psychol. Res. Behav. Manag..

[38] Brooks S.K., Webster R.K., Smith L.E., Woodland L., Wessely S., Greenberg N., Rubin G.J. (2020). The psychological impact of quarantine and how to reduce it: Rapid review of the evidence. Lancet.

[39] Sharp M.-L., Serfioti D., Jones M., Burdett H., Pernet D., Hull L., Murphy D., Wessely S., Fear N.T. (2021). UK veterans’ mental health and well-being before and during the COVID-19 pandemic: A longitudinal cohort study. BMJ Open.

[40] Yu J., Huang C., Han Z., He T., Li M. (2020). Investigating the Influence of Interaction on Learning Persistence in Online Settings: Moderation or Mediation of Academic Emotions?. Int. J. Environ. Res. Public Health.

[41] Yang X.-J., Liu Q.-Q., Lian S.-L., Zhou Z.-K. (2020). Are bored minds more likely to be addicted? The relationship between boredom proneness and problematic mobile phone use. Addict. Behav..

[42] Leung L. (2008). Linking psychological attributes to addiction and improper use of the mobile phone among adolescents in Hong Kong. J. Child. Media.

[43] Liu H., Wang H.L. (2012). Mobile phone addiction tendency and loneliness in college students. Chin. Ment. Health J..

[44] Huang H., Niu L., Zhou C., Wu H. (2014). Reliability and Validity of Mobile Phone Addiction Index for Chinese College Students. Chin. J. Clin. Psychol..

[45] Yang T., Huang H. (2003). An epidemiological study on stress among urban residents in social transition period. Chin. J. Epidemiol..

[46] Wang J.-L., Wang H.-Z., Gaskin J., Wang L.-H. (2015). The role of stress and motivation in problematic smartphone use among college students. Comput. Hum. Behav..

[47] Hunter J.A., Dyer K.J., Cribbie R.A., Eastwood J.D. (2016). Exploring the Utility of the Multidimensional State Boredom Scale. Eur. J. Psychol. Assess..

[48] Liu Y., Chen J., Jiang M., Xu H., Liu J., Eastwood J., Mehranvar S. (2013). The Chinese Version of the Multidimensional State Boredom Scale (MSBS): It’s Applicability in Chinese College Students. Chin. J. Clin. Psychol..

[49] Moshagen M., Erdfelder E. (2015). A New Strategy for Testing Structural Equation Models. Struct. Equ. Model. A Multidiscip. J..

[50] West S.G., Taylor A.B., Wu W., Hoyle R.H. (2012). Model fit and model selection in structural equation modeling. Handbook of Structural Equation Modeling.

[51] Podsakoff P.M., MacKenzie S.B., Lee J.-Y., Podsakoff N.P. (2003). Common method biases in behavioral research: A critical review of the literature and recommended remedies. J. Appl. Psychol..

[52] Shin Y.-J., Lee J.-Y. (2019). Self-Focused Attention and Career Anxiety: The Mediating Role of Career Adaptability. Career Dev. Q..

[53] Zhang Y., Tan D.-L., Lei T.-T. (2019). Parental Attachment and Problematic Smartphone Use Among Chinese Young Adults: A Moderated Mediation Model of Interpersonal Adaptation and Self-control. J. Adult Dev..

[54] Gusman M.S., Grimm K.J., Cohen A.B., Doane L.D. (2021). Stress and sleep across the onset of the novel coronavirus disease 2019 pandemic: Impact of distance learning on US college students’ health trajectories. Sleep.

[55] Serra G., Scalzo L.L., Giuffrè M., Ferrara P., Corsello G. (2021). Smartphone use and addiction during the coronavirus disease 2019 (COVID-19) pandemic: Cohort study on 184 Italian children and adolescents. Ital. J. Pediatr..

[56] Oka T., Hamamura T., Miyake Y., Kobayashi N., Honjo M., Kawato M., Kubo T., Chiba T. (2021). Prevalence and risk factors of internet gaming disorder and problematic internet use before and during the COVID-19 pandemic: A large online survey of Japanese adults. J. Psychiatr. Res..

[57] Fighting COVID-19: China in Action. http://english.www.gov.cn/news/topnews/202006/07/content_WS5edc559ac6d066592a449030.html.

[58] Zhang W., Zhou F., Zhang Q., Lyu Z. (2022). Attachment anxiety and smartphone addiction among university students during confinement: Teacher–student relationships, student–student relationships and school connectedness as mediators. Front. Public Health.

[59] Thomée S., Eklöf M., Gustafsson E., Nilsson R., Hagberg M. (2007). Prevalence of perceived stress, symptoms of depression and sleep disturbances in relation to information and communication technology (ICT) use among young adults—An explorative prospective study. Comput. Hum. Behav..

[60] Wegmann E., Ostendorf S., Brand M. (2018). Is it beneficial to use Internet-communication for escaping from boredom? Boredom proneness interacts with cue-induced craving and avoidance expectancies in explaining symptoms of Internet-communication disorder. PLoS ONE.

[61] Yang X., Yip B.H.K., Lee E.K.P., Zhang D., Wong S.Y.S. (2021). The Relationship Between Technology Use and Problem Technology Use and Potential Psychosocial Mechanisms: Population-Based Telephone Survey in Community Adults During COVID-19. Front. Psychol..

[62] Gabbiadini A., Baldissarri C., Durante F., Valtorta R.R., De Rosa M., Gallucci M. (2020). Together Apart: The Mitigating Role of Digital Communication Technologies on Negative Affect During the COVID-19 Outbreak in Italy. Front. Psychol..

[63] Olfert M.D., Wattick R.A., Saurborn E.G., Hagedorn R.L. (2022). Impact of COVID-19 on college student diet quality and physical activity. Nutr. Health.

